# Injection of colorectal cancer cells in mesenteric and antimesenteric sides of the colon results in different patterns of metastatic diffusion: An experimental study in rats

**DOI:** 10.1186/1477-7819-3-69

**Published:** 2005-10-19

**Authors:** Luigi Boni, Angelo Benevento, Gianlorenzo Dionigi, Francesca Rovera, Mario Diurni, Renzo Dionigi

**Affiliations:** 1 Department of Surgery, University of Insubria, Ospedale di Circolo e Fondazione Macchi, Varese, Italy

## Abstract

**Background:**

This experimental study was designed to investigate the differences in pattern of local growth and diffusion of colorectal cancer cells injected into either mesenteric (M) or antimesenteric (AM) sides of the colon.

**Methods:**

A total of 1 × 10^6 ^colonic adenocarcinoma cells (line DHD/K12-TRb) were injected into the cecal wall of BDIX syngeneic male rats at an M or AM site of the colon. At six weeks after injection, all animals were sacrificed and the presence or absence of tumor in the cecum as well as regional metastasis and peritoneal carcinomatosis were determined.

**Results:**

Six weeks after injection, macroscopic tumor growth was observed in 27/37 (72%) animals in group M and 21/32 (65%) in group AM (*P *= 0.98). In group AM, diffuse peritoneal carcinomatosis was present in 19/21 rats (90.4%) versus 3/27 rats (11%) in group M; this difference was statistically significant (*P *= 0.025). Regional mesenteric lymph nodes were the only location in which tumor was detected in 23/27 rats (85%) in group M versus 2/21 (9.5%) in group AM; this difference too was statistically significant (*P *= 0.031)

**Conclusion:**

The patterns of diffusion of tumors implanted in mesenteric and antimesenteric sites of the colon appear to be different, although the reason for this is not clear.

## Background

Colorectal cancer is the second leading cause of death from tumors in the world with a worldwide incidence of more than 1 million cases [1]. As for most malignant tumors, the degree of infiltration of the colonic wall (T stage) and presence of lymph node/ distant (N and M stage) metastases have been demonstrated to be strongly related to prognosis [2]. Identifying the pattern of cancer spread (local and distant) may be important in choosing the appropriate surgical and medical, strategies in relation to various factors, such as tumor location and site of growth.

Several experimental models have been developed to examine colorectal cancer kinetics, but most are based on heterotopic cell implantation (i.e. peritoneal, subcutaneous, etc.) [3,4] both in nude and syngeneic rats [5,6].

Other investigators have developed orthotopic models in which tumor implantation is performed directly into the wall of the colon of healthy rats, in order to study patterns of local growth and metastatic spread [7,8].

The present experimental study was designed to investigate the differences in pattern of local growth and diffusion of colorectal cancer cells injected into either mesenteric (M) or antimesenteric (AM) sides of the colon. Such differences may be of clinical importance, since metastases and diffusion could be influenced by tumor location.

## Materials and methods

### Animals

BDIX syngeneic male rats (Charles Rivers Italia – Lecco, Italy) with a mean weight of 290 ± 15 g were used for all experiments. They were kept in protective cages with controlled air in/outflow and allowed free access to food and water. They were divided into two groups, mesenteric (M) and antimesenteric (AM), of 40 rats each.

### Tumor cell line

The colonic adenocarcinoma cell line DHD/K12-TRb (European Collection of Cell Culture, Salisbury, Wiltshire – UK) was used in all experiments. These cells were cloned [3] from a 1,2-dimethylhydrazine-induced colonic adenocarcinoma and maintained in monolayers using DMEM medium enriched with Ham's F10 (1:1, v/v; GIBCO, Life Technologies, Paisley, UK) supplemented with 10% fetal bovine serum (GIBCO) and 0.005% gentamycin (GIBCO). The cells were divided every 72 hours after dispersion in 0.125% EDTA-trypsin.

Prior to injection, cells viability was confirmed by trypan-blue test, and was found to be greater than 95% in all cases.

### Tumor implantation model and experimental procedure

**Figure 1:**
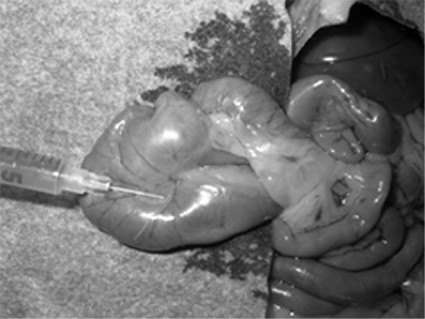
Intraparietal injection of colonic cancer cells (mesenteric injection).

Each animal was anaesthetized by intraperitoneal injection of 75 mg/kg of ketamine (Sigma-Aldrigh, Italy). A 2 cm midline laparotomy was performed and the cecum was exposed. Intraparietal injection (Fig. 1) of 1 × 10^6 ^cells in 0.25 ml of buffer solution was performed in a mesenteric (Group M) or antimesenteric (Group AM) side of the cecum (Figure 1).

Extreme care was taken to avoid accidental spillage of cells into the abdominal cavity or tumor injection directly into the bowel lumen: the creation of a macroscopically visible blister in the bowel wall was required for confirmation of intra-parietal injection. The laparotomy was closed with reabsorbable 3/0 suture.

**Figure 2:**
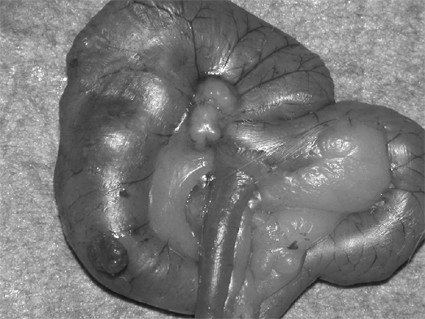
Mesenteric growth of implanted colonic cancer.

**Figure 3:**
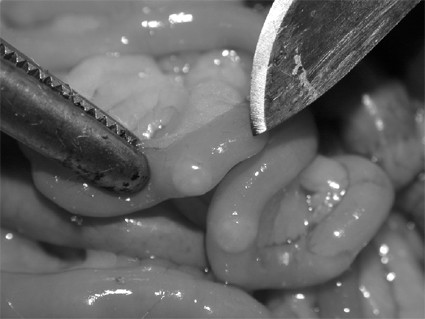
Peritoneal carcinomatosis after antimesenteric implantation of cancer cells.

At six week after injection, all animals were sacrificed with a lethal dose of sodium thiopental (Sigma-Aldrigh, Italy) A wide xiphopubic incision was performed and the presence or absence of tumor in the cecum as well as regional metastasis (Figure 2) and peritoneal carcinomatosis (Figure 3) was recorded. Histopathological examination of the primary tumor and metastases was always performed to confirm the features of the specimen.

### Statistical analysis

Student's t-test was used to compare numbers of tumor lesions and the characteristics of diffusion, with *P*-values less than 0.05 considered significant.

## Results

Three animals in group M and 8 in group AM died due to cardiorespiratory arrest during anesthesia or immediately after the procedure. Six weeks after tumor cell injection, macroscopic tumor growth was observed in 27/37 (72%) animals in group M and 21/32 (65%) in group AM; this difference was not significant (*P *= 0.98).

**Figure 4:**
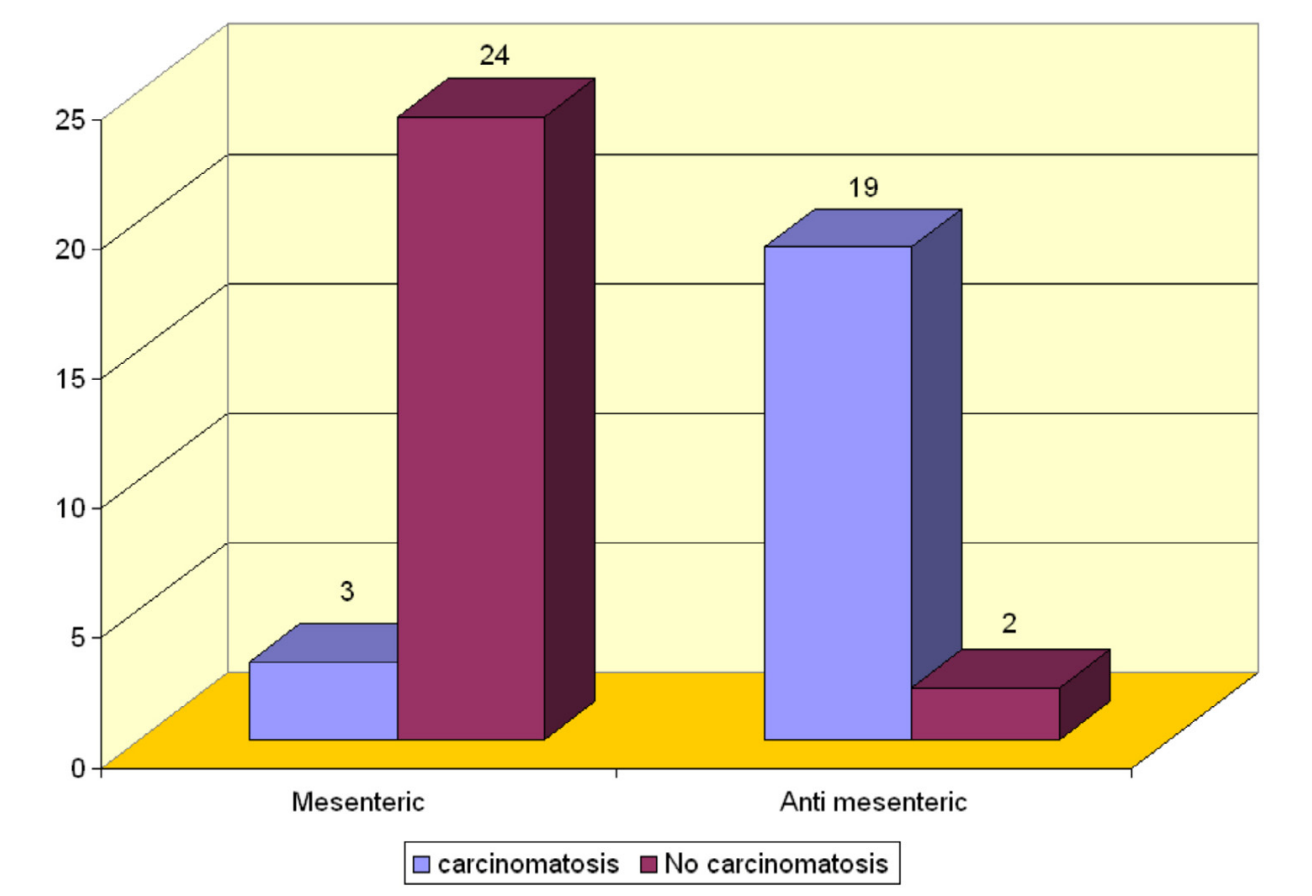
Incidence of peritoneal carcinomatosis in groups M and AM (*P *= 0.025).

In group AM, diffuse peritoneal carcinomatosis (Figure 3) was present in 19/21 rats (90.4%) versus 3/27 cases (11%) in group M (Figure 4); this difference was statistically significant (*P *= 0.025).

**Figure 5:**
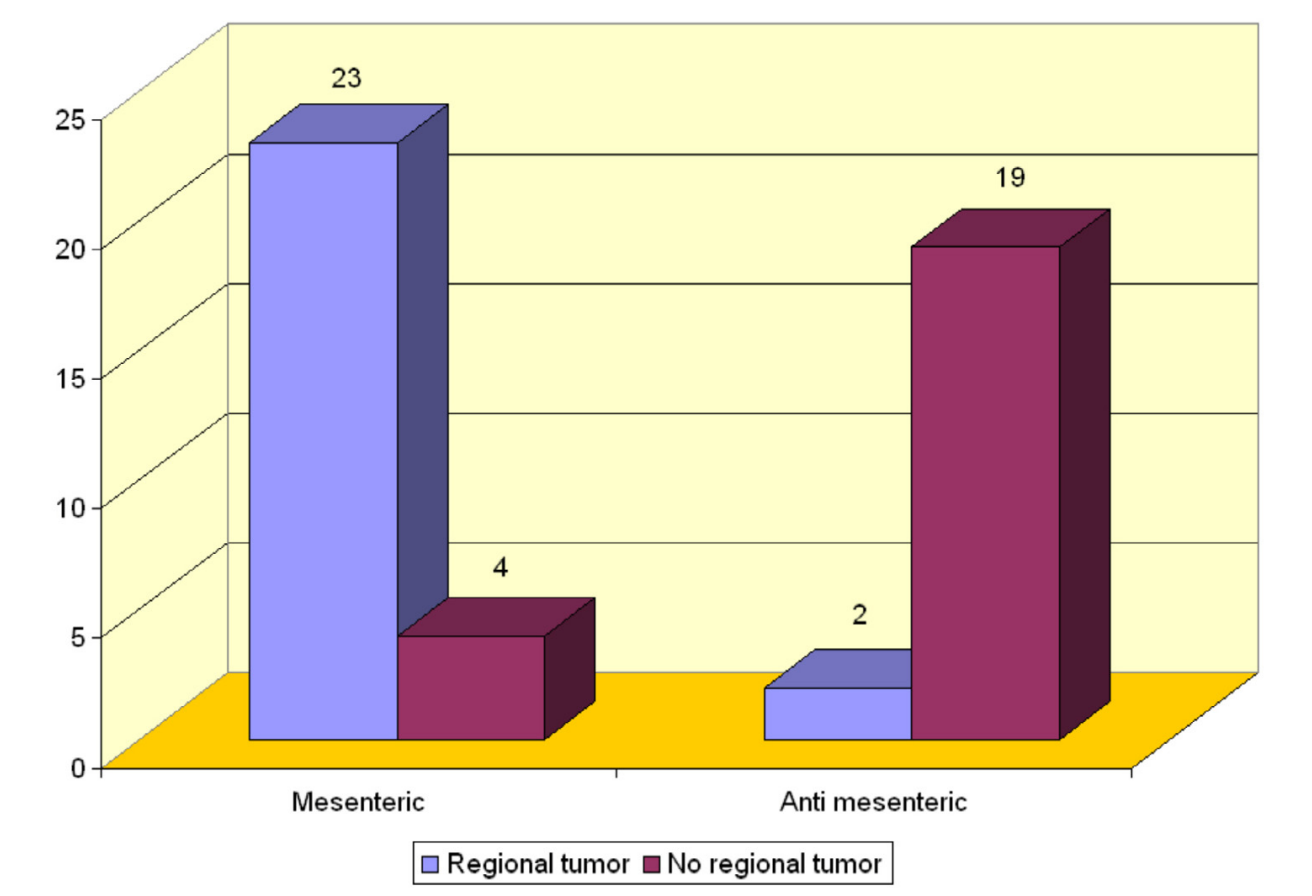
Incidence of mesenteric growth in groups M and AM (*P *= 0.031).

Regional mesenteric lymph nodes (Figure 2) (located near tumor growth) were the only manifestation of tumor diffusion in 23/27 rats (85%) in group M versus 2/21 (9.5%) in group AM (Figure 5); this difference was significant (*P *= 0.031). No liver or lung metastases were observed in either group. Histological confirmation of the presence of cancer cells was obtained in all cases.

## Discussion

Study of the kinetics of local growth and metastatic diffusion of cancers is extremely important for increasing knowledge of the natural history of the tumors and for determination of appropriate surgical strategies and/or medical therapies.

Although experimental models are the best means for study of tumor kinetics, exposure of animals to carcinogenic agents results in excessive variability such as differences in differentiation, location and diffusion. On the contrary, implantation of cancer cells or solid tumor, directly into the site to be studied, it can reproduce a standard condition more suitable for this kind of studies [3-7].

Several experimental models of free cancer cell implantation into the wall of the colon have been described [3,4]. The animal model used in our experiments is well-established, since it has been fully demonstrated that DHD/K12/TRb colonic cancer cells are able to grow when injected subcutaneously, intraperitoneally and in the wall of the colon [3-5]. Nevertheless, some of these studies used nude animals, in which unusual immunological status may result in great difficulty in interpretation of results.

In our study, the incidence of tumor growth after implantation was almost 65%; similar results have been reported by other studies [4,7]. Incorrect parietal injection, low viability of tumor cells, intraluminar injection, or host immune response to cancer cells may be explanations of the 65% tumor growth incidence.

Garcia Almo *et al*, [7], injecting DHD/K12/TRb cells into the cecal wall of syngeneic rats, demonstrated progressive tumor growth from stage T1 to stage T4, as well as lymph node involvement and distant metastases, reproducing the same progressive development as occurs in human colon cancer. Nevertheless, with this kind of injection model, it seems to be extremely difficult to perform precise implantation into the mucosa and to enable progressive tumor growth corresponding to that observed clinically.

In order to avoid accidental spillage of tumor cells and induce true local growth, Balague *et al*, [8] developed a model in which solid tumors, derived from DHD/K12/TRb colonic cancer cells, were implanted into the wall of the colon. Nevertheless, variability in the number of viable cells contained in a single piece of implanted tumor may result in misinterpretation of results.

None of the above studies considered difference in tumor implantation side, mesenteric or antimesenteric, as affecting local growth and distant metastases.

This difference might be of clinical significance, since certain locations can result in more aggressive tumor behavior due to particular anatomical patterns of blood or lymphatic vessels. In our study, when injection of tumor cells was performed in the mesenteric site of the colon, cancer grew locally and spread to the regional mesenteric lymph nodes. On the other hand, with antimesenteric implantation, there was lymph node metastasis but a high incidence of peritoneal carcinomatosis.

Proximity to larger blood and lymphatic vessels of the mesenteric site of the colon could explain the tendency of implanted cells to spread to regional nodes. When cancer cells are injected into the antimesenteric wall, peritoneal diffusion appears to be promoted, probably due to direct contact of the tumor with loops of bowel and the peritoneum.

Another explanation may be related to differences in microvascular pattern of the wall of the colon. Preliminary results of an experimental vascular casting study that we are performing in collaboration with the Department of Human Anatomy, seem to support the theory that the antimesenteric side of the colon differs significantly from the mesenteric side in microvascular arterial density and distribution (unpublished data).

Since M and N stage are the most important prognostic factors in human colorectal cancer [2], differences in local and metastatic diffusion of cancers located on the mesenteric or antimesenteric sites of the colon may be of clinical importance [9].

## Conclusion

The experimental model used in this study is able to induce local growth as well as peritoneal carcinomatosis in rats by parietal injection of malignant cells. The pattern of diffusion of tumors implanted at mesenteric and antimesenteric sites of the colon appear to differ, although the reason for this difference is unclear. Proximity of larger blood and lymphatic vessels or differences in vascular pattern in the wall of the colon between mesenteric and antimesenteric sites might account for these findings. Further clinical studies are needed to confirm the prognostic significance of the location of colorectal cancer in humans.

## Competing interests

The author(s) declare that they have no competing interests.

## Authors' contributions

**LB: **Clinical Lecturer in Surgery, Research leader he performed the experimental procedure and He wrote the experimental part of the manuscript

**AB: **Professor of Surgery, research project advisor, he supervised the experimental procedures and helped during the manuscript preparation

**GD: **Clinical Lecturer, assisted during the experimental procedure, he helped during the manuscript preparation (introduction and Discussion) and literature review

**FR: **Clinical Lecturer, She helped during the manuscript preparation especially in the statistical methods and discussion

**MD: **Staff surgeon, assisted during the experimental procedure

**RD: **Professor of Surgery, research project advisor, he helped during the manuscript preparation and final revision

All authors read and approved the final manuscript for publication.
